# Plasma microbial cell-free DNA metagenomic sequencing for early detection of infection after liver transplantation: a prospective cohort study

**DOI:** 10.3389/fcimb.2026.1930701

**Published:** 2026-10-15

**Authors:** Yongseop Lee, Eun-Ki Min, Yu Jin Park, Deok-Gie Kim, Dong Jin Joo, Myoung Soo Kim, Saeam Shin, Seung-Tae Lee, Jae Geun Lee, Su Jin Jeong

**Affiliations:** 1 Division of Infectious Diseases, Department of Internal Medicine and AIDS Research Institute, Yonsei University College of Medicine, Seoul, Republic of Korea; 2 Department of Surgery, Yonsei University College of Medicine, Seoul, Republic of Korea; 3 The Research Institute for Transplantation, Yonsei University College of Medicine, Seoul, Republic of Korea; 4 Department of Laboratory Medicine, Yonsei University College of Medicine, Seoul, Republic of Korea

**Keywords:** bloodstream infection, infection diagnosis, liver transplantation, metagenomic next-generation sequencing, microbial cell-free DNA

## Abstract

**Introduction:**

Microbial cell-free DNA (mcfDNA) metagenomic next-generation sequencing (mNGS) is a promising technique for diagnosing infectious diseases. However, research on mcfDNA-based mNGS for diagnosing infections in liver transplantation (LT) recipients remains limited. The aim of this study was to evaluate the diagnostic performance and clinical utility of plasma mcfDNA mNGS during the early post-LT period.

**Methods:**

In this prospective diagnostic cohort study, adult LT recipients underwent plasma mcfDNA mNGS on postoperative days 7 and 14. The findings were compared with results of the blood cultures and other conventional microbiological tests at the species level.

**Results:**

Among 193 LT recipients, mNGS positivity was observed in 14.5% on day 7 and 21.8% on day 14. The concordance between mNGS and blood culture for bacterial pathogens was modest on days 7 (35.0%) and 14 (14.3%) but increased substantially when other clinically relevant specimens were included (80.0% and 71.4%, respectively). mNGS identified additional pathogens not detected using blood culture in 59.3% and 74.2% of cases on days 7 and 14, respectively. Viral pathogens, including cytomegalovirus, herpes simplex virus, varicella-zoster virus, and roseolovirus, were also detected. Among the 13 discordant cases (blood culture-positive and mNGS-negative), two (15.4%) were classified as contamination/colonization, five (38.5%) as weak positives, and six (46.2%) as not detected by mNGS.

**Conclusion:**

Plasma mcfDNA mNGS demonstrated substantial concordance with conventional microbiological testing when clinically relevant specimens were considered. mNGS may serve as a useful adjunct tool for early pathogen detection and clinical assessment of LT recipients.

## 1 Introduction

Infection-related complications after liver transplantation (LT) are common and are a major cause of mortality. Bacterial infections occur in approximately 20–80% of LT recipients, and opportunistic infections are a leading cause of death during the first three years after LT (Romero and Razonable, 2011). However, diagnosing infections during the early post-transplantation period is often challenging. Immunosuppressive therapy attenuates inflammatory responses, which may blunt or mask clinical signs of infection and delay diagnosis and treatment (Romero and Razonable, 2011). Furthermore, opportunistic infections caused by atypical pathogens may occur, and non-infectious complications such as postoperative bleeding or rejection can mimic infectious disease (Hernandez Mdel et al., 2015).

Microbial cell-free DNA (mcfDNA) metagenomic next-generation sequencing (mNGS) is a promising technique for diagnosing infectious diseases and may overcome the limitations of conventional diagnostic methods in LT recipients. mcfDNA-based mNGS can detect causative pathogens with higher sensitivity than conventional culture-based methods, particularly in patients who have received broad-spectrum empirical antibiotics (Li et al., 2024; Miao et al., 2018; Wilson et al., 2019). The hypothesis- and culture-independent nature of mNGS enables detection of rare, novel, or fastidious microorganisms (Elbehiry and Abalkhail, 2025). Moreover, cell-free DNA (cfDNA)-based assays can help distinguish accompanying clinical conditions in LT recipients, including graft injury, acute rejection, graft-versus-host disease, and the recurrence of hepatocellular carcinoma (Avramidou et al., 2023).

Nevertheless, studies evaluating the use of mcfDNA-based mNGS for diagnosing infections in LT recipients remain limited. In this study, we aimed to evaluate the diagnostic accuracy of mcfDNA mNGS during the early post-LT period compared with conventional diagnostic methods for detecting bloodstream infections. Additionally, we analyzed cases of discordance between mcfDNA mNGS and standard tests to determine the additional clinical utility of mcfDNA mNGS. Our findings may support the clinical utility of mcfDNA mNGS for improving early diagnosis of infections after LT and complementing conventional methods.

## 2 Methods

### 2.1 Study design and population

This prospective diagnostic cohort study was conducted between January 2024 and June 2025 at Severance Hospital, a large tertiary teaching hospital in South Korea. This study was conducted as part of a cfDNA-based diagnostic platform designed to detect acute rejection, graft injury, hepatocellular carcinoma recurrence, and infection, in accordance with the ethical guidelines of the Declaration of Helsinki and Istanbul. The study was approved by the Severance Hospital Institutional Review Board (approval number 4-2023-1012). All participants provided written informed consent before surgery.

Patients aged ≥18 years who underwent LT were eligible for inclusion. The participants underwent mcfDNA mNGS on postoperative days 7 and 14. The test results were provided to the clinical team and incorporated into patient management when deemed necessary. Patients were excluded from the analysis if a cfDNA sample was not obtained on day 7 or 14. Participants were followed up until death or loss to follow-up. The data cut-off date was set to October 1, 2025, allowing a 90-day mortality assessment. LT and immunosuppression were performed in accordance with the institutional standard protocol (**Supplementary Methods S1**) (Choi et al., 2024).

### 2.2 mcfDNA mNGS procedures

Whole blood (9 or 18 mL) was collected in Streck Cell-Free DNA BCT tubes (Streck, Omaha, NE, USA) and centrifuged twice at 1900 ×*g* to separate the plasma. cfDNA was extracted using a magnetic circulating DNA Maxi Reagent kit (Dxome, Seongnam, Republic of Korea) and quantified using a Qubit HS dsDNA assay (Invitrogen, Carlsbad, CA, USA). Libraries were prepared and sequenced on an Illumina NovaSeq 6000 system (Illumina, San Diego, CA, USA) with 2 × 151 bp reads at ~5× depth. Reads were aligned to the human genome (GRCh37) using BWA (v0.7.17-r1188) (Li and Durbin, 2009), and unmapped reads were used for metagenomic analysis using Kraken2 (v2.1.2) (Wood et al., 2019). A custom Kraken2 database built from NCBI bacterial, viral, and fungal genomes enabled taxonomic classification (Federhen, 2012). To normalize the sequences across samples with varying sequencing depths, read counts were converted into reads per ten million (RPTM). A uniform threshold of RPTM ≥ 5 was applied to all microorganisms to define a positive detection. Potential contaminants were mitigated by masking known environmental and skin flora, such as *Cutibacterium acnes* and *Burkholderia contaminans*, and manually excluding organisms with high abundance restricted to specific processing batches, indicating contamination during DNA extraction or library preparation.

### 2.3 Cross-validation of fungal detection in mcfDNA mNGS

To address potential database-driven false discoveries and assess the sensitivity of the primary pipeline for fungal surveillance, Kraken2 results were cross-validated using two auxiliary classifiers. Kaiju (v1.9.0) (Menzel et al., 2016) uses translated protein matching, whereas EukDetect (v1.2) (Lind and Pollard, 2021) uses marker-gene alignment. To establish an alignment-based reference for presence or absence, supporting reads were retrieved from host-unmapped FASTQ files using seqtk (v1.3) (Li, 2012) and mapped with BWA-MEM (v0.7.17) (Li and Durbin, 2009) against a custom reference panel comprising 416 fungal genomes retrieved from NCBI, supplemented with the human GRCh38 genome as a decoy. Candidate calls were considered confirmed when supported by ≥10 reads with MAPQ ≥30 distributed across ≥3 distinct 1-kb windows; otherwise, they were considered refuted.

### 2.4 Conventional diagnostic tests

Routine cultures (two sets of peripheral blood, sputum, peritoneal drain, urine, and stool) were performed on postoperative days 0, 1, and 2 during the intensive care unit (ICU) stay, and every other day thereafter if ICU care continued. During general ward care, blood cultures were performed as indicated. The blood culture bottles were incubated at 37 °C for 5 days, and the organisms were identified using a matrix-assisted laser desorption/ionization time-of-flight mass spectrometry system (Bruker Daltonics, Bremen, Germany).

Routine cytomegalovirus (CMV) polymerase chain reaction (PCR) testing was conducted on postoperative days 7 and 14, and subsequently on a weekly basis for up to 1 month postoperatively during continued hospitalization. Plasma samples were used for CMV quantification. CMV DNA levels were measured using a fully automated Cobas 6800 system (Roche Molecular Systems, Branchburg, NJ, USA) with the Cobas CMV Quantitative Nucleic Acid Test.

### 2.5 Variables and outcome measures

This study also used data from a previously established LT cohort described in a previous study (Lee et al., 2025). Baseline demographic, clinical, and transplantation-related variables were prospectively collected. Laboratory and clinical severity parameters at the time of cfDNA sampling were analyzed. No additional tests were performed for research purposes, and cases without available measurements were treated as having missing data. The outcomes of interest were 28- and 90-day mortality. When viral pathogens other than CMV were detected using mNGS, additional variables such as antiviral treatment and skin manifestations suggestive of viral infection were collected retrospectively.

### 2.6 Concordance between mNGS and conventional diagnostic tests

Cases wherein both mNGS and blood culture yielded positive results were considered double positive and classified as matched, partially matched, or mismatched at the species level. Cases in which only the blood culture was positive were considered culture-positive, and those in which only mNGS was positive were considered mNGS-positive. Cases with negative results in both tests were considered double negative. When mNGS was negative and a blood culture was not performed, the result was considered mNGS-negative.

Additional analyses incorporating culture results from other clinically relevant specimens within 14 days were performed to assess possible concordance (Smith et al., 2022). Discordant cases were further classified as contamination, colonization, weakly positive, or false negative based on predefined criteria (Hall and Lyman, 2006; Mermel et al., 2009). Contamination was defined as organisms considered skin contaminants isolated from a single bottle of a blood culture pair. Colonization was defined as commensal skin flora isolated solely from a central venous catheter specimen and not from a peripheral blood culture. When mNGS detected the organism below the predefined threshold, the finding was interpreted as weakly positive. All remaining cases were classified as false negatives. Detailed definitions are provided in **Supplementary Methods S2**.

### 2.7 Statistical analysis

Continuous variables are presented as mean ± standard deviation for normally distributed data and as median with interquartile ranges for non-normally distributed data. Normality was assessed using the Shapiro–Wilk test. Between-group comparisons of continuous variables were performed using the independent two-tailed t-test or Mann–Whitney U test, as appropriate. Categorical variables are expressed as number (%) and were compared between the groups using Pearson’s chi-squared test or Fisher’s exact test, as appropriate. Statistical significance was defined at a two-sided *P*-value < 0.05. Statistical analyses were performed using R (version 4.4.0, The R Foundation for Statistical Computing, Vienna, Austria).

## 3 Results

### 3.1 Patient enrollment and baseline characteristics

In total, 216 LT recipients consented to participate in this study (**Figure 1**). Of these, 23 patients were excluded because cfDNA sampling was not performed on either post-LT day 7 or 14, resulting in 193 participants included in the final analysis. cfDNA sampling was performed on post-LT days 7 and 14 in 179 and 174 patients, respectively, and 160 patients were sampled at both time points.

**Figure 1:**
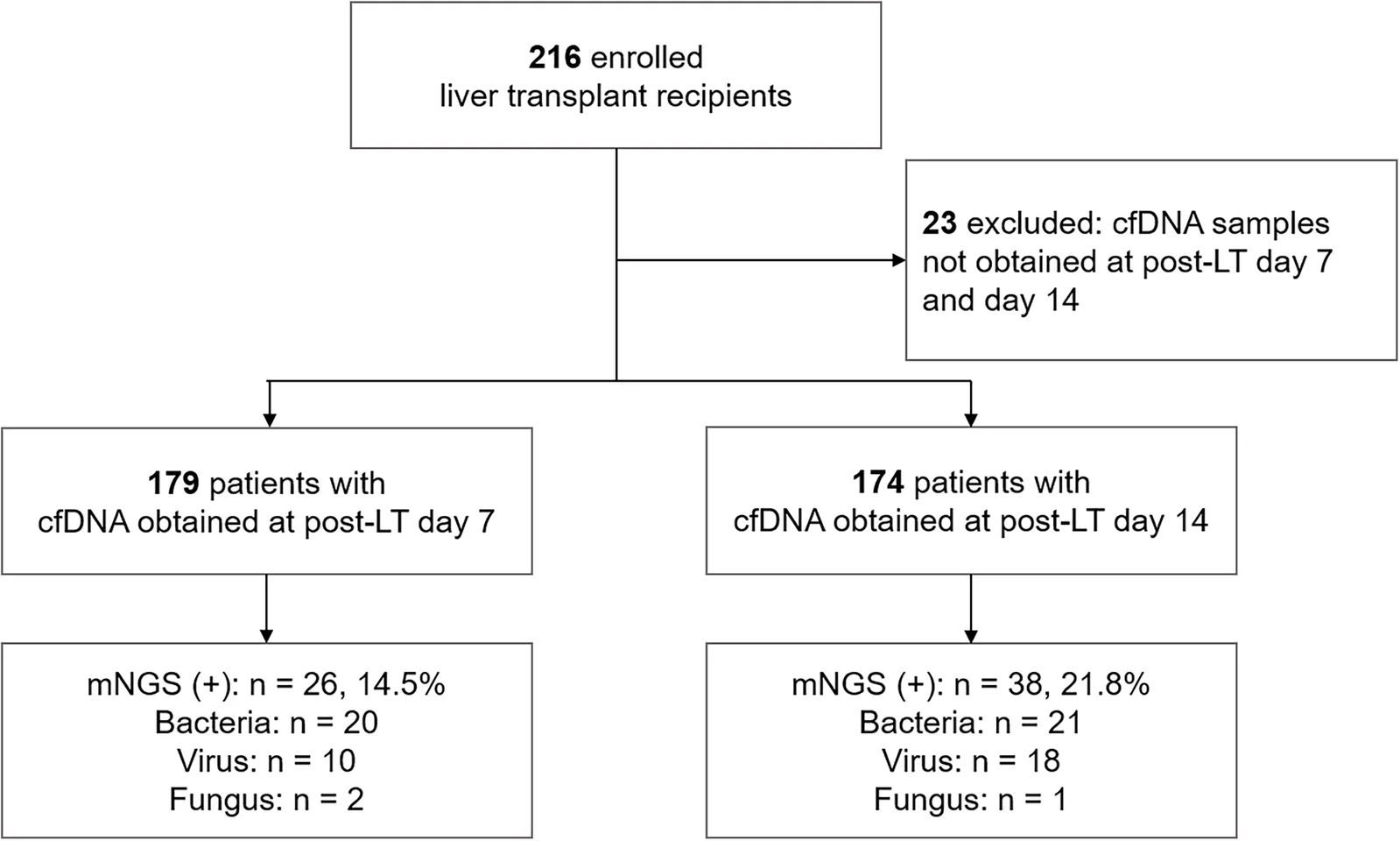
Flowchart of participant enrollment. cfDNA, cell-free DNA; LT, liver transplantation; mNGS, metagenomic next-generation sequencing.

mNGS positivity was identified in 26 (14.5%) and 38 (21.8%) patients on days 7 and 14, respectively (**Table 1**). Baseline characteristics were similar between the groups, except for more impaired pre-transplant liver function and a higher frequency of deceased donor LT in the mNGS(+) group than in the mNGS(–) group. The laboratory findings obtained at the time of mNGS testing were compared based on mNGS positivity. On day 7, the mNGS(+) group had a significantly higher median (interquartile range) cfDNA concentration than did the mNGS(–) group (54, 560 [32, 835–138, 030] vs. 35, 475 pg/mL [25, 155–52, 890]; *P* = 0.006; **Table 1**). Total bilirubin levels were significantly higher in the mNGS(+) group than in the mNGS(–) group at both time points.

**Table 1:** Comparison of baseline characteristics and laboratory findings of liver transplantation recipients according to mNGS results.

	Post-LT, Day 7			Post-LT, Day 14		
Variable	mNGS(–) (n = 153)	mNGS(+) (n = 26)	*P*-value	mNGS(–) (n = 136)	mNGS(+) (n = 38)	*P*-value
Age, years	56.0 (50.0–63.0)	56.5 (51.0–62.0)	0.971	56.0 (50.0–62.5)	56.5 (51.0–62.0)	0.597
Male sex	95 (62.1)	14 (53.8)	0.426	85 (62.5)	24 (63.2)	0.941
Hypertension	36 (23.5)	5 (19.2)	0.630	31 (22.8)	9 (23.7)	0.908
DM	55 (35.9)	8 (30.8)	0.609	44 (32.4)	18 (47.4)	0.088
CVD	12 (7.8)	3 (11.5)	0.530	8 (5.9)	6 (15.8)	0.047
Underlying liver diseases			0.901			0.775
Viral	66 (43.1)	10 (38.5)		62 (45.6)	15 (39.5)	
Alcoholic	50 (32.7)	9 (34.6)		43 (31.6)	14 (36.8)	
Others	37 (24.2)	7 (26.9)		31 (22.8)	9 (23.7)	
HCC	51 (33.3)	9 (34.6)	0.898	46 (33.8)	15 (39.5)	0.519
CTP score			0.076			0.001
A	43 (28.1)	4 (15.4)		44 (32.4)	2 (5.3)	
B	57 (37.3)	7 (26.9)		51 (37.5)	15 (39.5)	
C	53 (34.6)	15 (57.7)		41 (30.1)	21 (55.3)	
MELD score	16.0 (11.0–23.0)	26.0 (14.0–37.0)	0.008	15.0 (11.0–23.0)	28.0 (17.0–38.0)	<0.001
Re-transplantation	3 (2.0)	3 (11.5)	0.012	1 (0.7)	5 (13.2)	<0.001
Combined transplantation	1 (0.7)	0 (0.0)	0.679	1 (0.7)	1 (2.6)	0.332
DDLT	18 (11.8)	8 (30.8)	0.011	14 (10.3)	13 (34.2)	<0.001
ABO incompatibility	37 (24.2)	3 (11.5)	0.152	34 (25.0)	6 (15.8)	0.233
GRWR	1.1 (0.9–1.4)	1.1 (0.8–1.3)	0.821	1.0 (0.9–1.4)	1.2 (1.0–1.7)	0.021
GRWR < 0.8	22 (14.4)	3 (12.0)	0.751	19 (14.0)	4 (10.8)	0.616
RBC transfusion, pack	6.0 (3.0–9.0)	7.0 (6.0–11.0)	0.043	5.0 (3.0–9.0)	8.0 (6.0–11.0)	0.004
Splenectomy	23 (15.0)	6 (23.1)	0.303	19 (14.0)	7 (18.4)	0.496
Steroid pulse therapy within 14 days	–	–	–	18 (13.2)	5 (13.2)	0.990
cfDNA level, pg/mL^a)^	35, 475.0 (25, 155.0–52, 890.0)	54, 560.0 (32, 835.0–138, 030.0)	0.006	26, 257.5 (18, 189.0–43, 215.0)	32, 775.0 (20, 955.0–51, 600.0)	0.220
AST level, IU/L^a)^	89.0 (53.0–130.0)	98.5 (58.0–156.0)	0.475	27.5 (17.0–47.5)	26.0 (17.0–39.0)	0.596
ALT level, IU/L^a)^	45.0 (33.0–68.0)	53.0 (38.0–78.0)	0.162	34.0 (23.5–55.5)	32.5 (22.0–69.0)	0.783
T. bilirubin level, mg/dL^a)^	2.5 (1.6–4.9)	5.7 (2.0–9.8)	0.006	1.3 (1.0–2.2)	3.6 (1.5–5.0)	<0.001
Prothrombin time, INR ^a)^	1.1 (1.0–1.3)	1.1 (1.0–1.3)	0.538	1.1 (1.0–1.1)	1.1 (1.0–1.2)	0.228
CRP level (mg/L)	10.8 (5.4–23.2)	39.6 (14.2–87.3)	<0.001	15.5 (5.0–32.7)	57.0 (20.5–104.2)	<0.001
IgG level, mg/dL^a)^	614.0 (473.0–819.0)	753.0 (567.0–940.0)	0.140	561.0 (390.0–816.0)	596.0 (433.0–872.0)	0.348
IgG level < 700 mg/dL	90 (60.0)	11 (47.8)	0.270	80 (62.0)	22 (61.1)	0.921
28-day mortality	0 (0.0)	7 (26.9)	<0.001	2 (1.5)	2 (5.3)	0.168
90-day mortality	6 (3.9)	8 (30.8)	<0.001	4 (2.9)	5 (13.2)	0.012

a) Variables were measured on post-LT days 7 and 14, and values are presented according to the timing of mNGS collection.

mNGS, metagenomic next-generation sequencing; LT, liver transplantation; DM, diabetes mellitus; CVD, cardiovascular disease; HCC, hepatocellular carcinoma; CTP, Child–Pugh; MELD, Model for End-stage Liver Disease; DDLT, deceased-donor liver transplantation; GRWR, graft-to-recipient weight ratio; RBC, red blood cell; cfDNA, cell-free DNA; AST, aspartate aminotransferase; ALT, alanine aminotransferase; INR, international normalized ratio; CRP, C-reactive protein; IgG, immunoglobulin G; T., total.

On day 7, the mNGS(+) group had higher 28- and 90-day mortality rates than the mNGS(–) group. On day 14, the 28-day mortality rate did not differ significantly according to mNGS positivity; however, the 90-day mortality rate remained significantly higher in the mNGS(+) group.

### 3.2 Concordance between mcfDNA mNGS and conventional diagnostic tests

On day 7, 11 patients (6.1%) were double positive, with 9 matches and 2 mismatches (**Figure 2A**). mNGS(+) alone was observed in 15 (8.4%) patients, whereas culture(+) alone was identified in 5 (2.8%). Double-negative was observed in 65 (36.3%) cases and mNGS(–) in 83 (46.4%). mNGS(–) category refers to cases in which a culture was not performed at the time of mNGS testing, limiting the comparison with the culture results.

**Figure 2:**
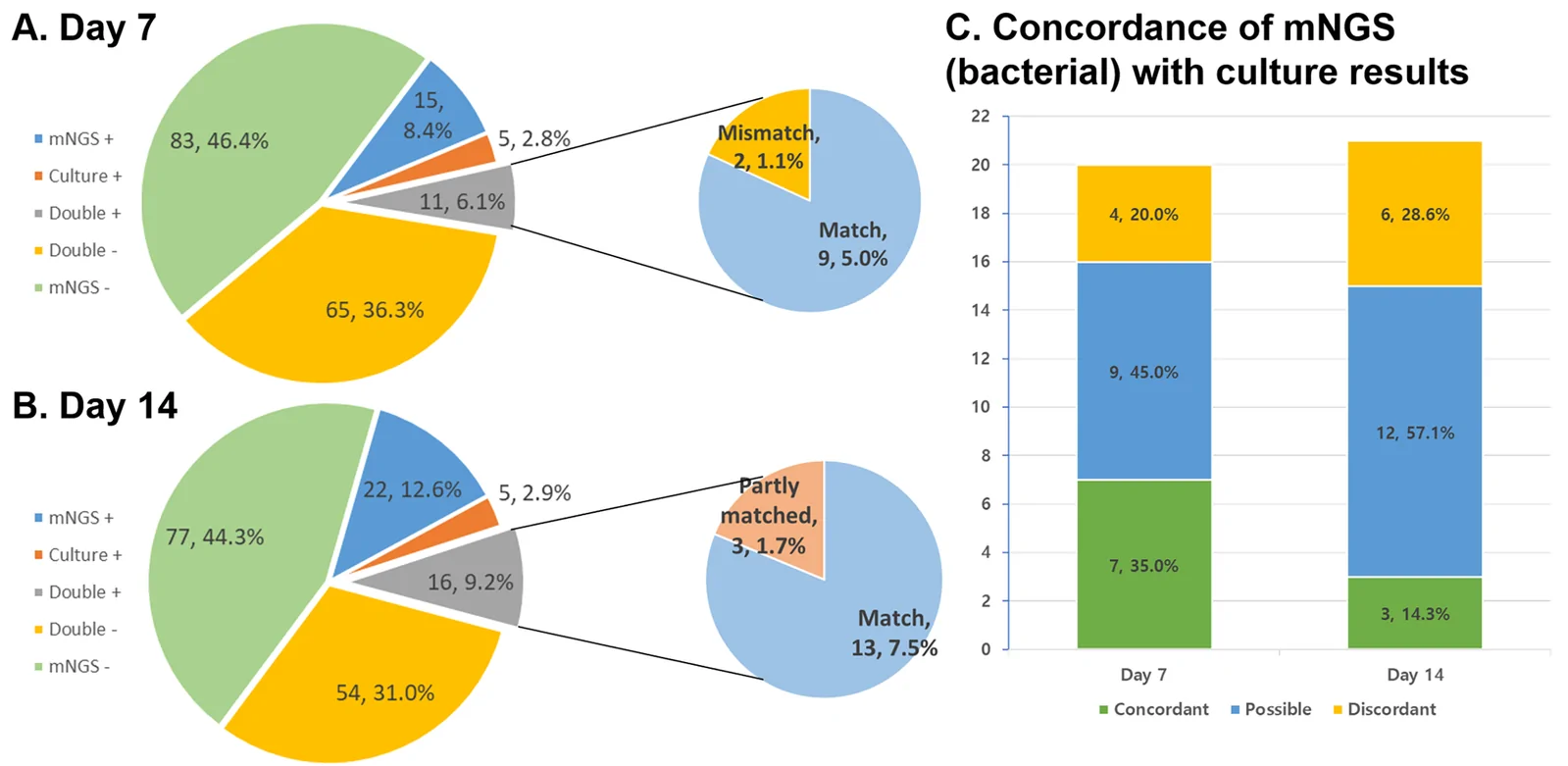
Concordance between mNGS and bloodstream infection diagnosis in liver transplantation recipients. **(A)** Results on day 7 post-LT. **(B)** Results on day 14 post-LT. **(C)** Concordance between bacterial pathogens detected using mNGS and blood culture. Possible bacteremia is determined by comparison with culture results from other clinically significant specimens in addition to blood cultures. The mNGS-negative category indicates cases in which a culture was not performed at the time of mNGS testing, making culture results unavailable. mNGS, metagenomic next-generation sequencing; LT, liver transplantation.

On day 14, 16 patients (9.2%) were double(+), with 13 (7.5%) cases showing full matching and 3 (1.7%) showing partial matching (**Figure 2B**). mNGS(+) alone was observed in 22 (12.6%) patients, whereas culture(+) alone was identified in 5 (2.9%). Double(–) and mNGS(–) accounted for 54 (31.0%) and 77 (44.3%) patients, respectively.

Among the cases with bacterial pathogens identified using mNGS, the concurrent blood cultures confirmed the presence of the same organism in seven (35.0%) and three cases (14.3%) on days 7 and 14, respectively (**Figure 2C**). When the culture results from other clinically significant specimens were included, overall concordance increased to 80.0% and 71.4% on days 7 and 14, respectively.

**Supplementary Tables S1** and **S2** summarize the detailed clinical characteristics and the likelihood of bacteremia among patients with positive mNGS results but negative culture results on days 7 and 14, respectively. Most patients received active antibiotics against the pathogen identified using mNGS at the time of testing (day 7, 84.6%; day 14, 94.1%).

### 3.3 Distribution of microorganisms detected using mNGS and blood culture

On days 7 and 14, 27 and 31 bacterial pathogens were identified using mNGS and blood cultures, respectively (**Figure 3**). Fungal pathogens were detected in 5 and 4 cases, and viral pathogens were detected in 9 and 17 cases on days 7 and 14, respectively. Among the bacterial pathogens detected using mNGS alone, 16 of 27 cases (59.3%) were detected on day 7 and 23 of 31 cases (74.2%) on day 14. For fungi, only one additional pathogen, *Aspergillus* species, detected on day 7, was exclusively identified using mNGS.

**Figure 3:**
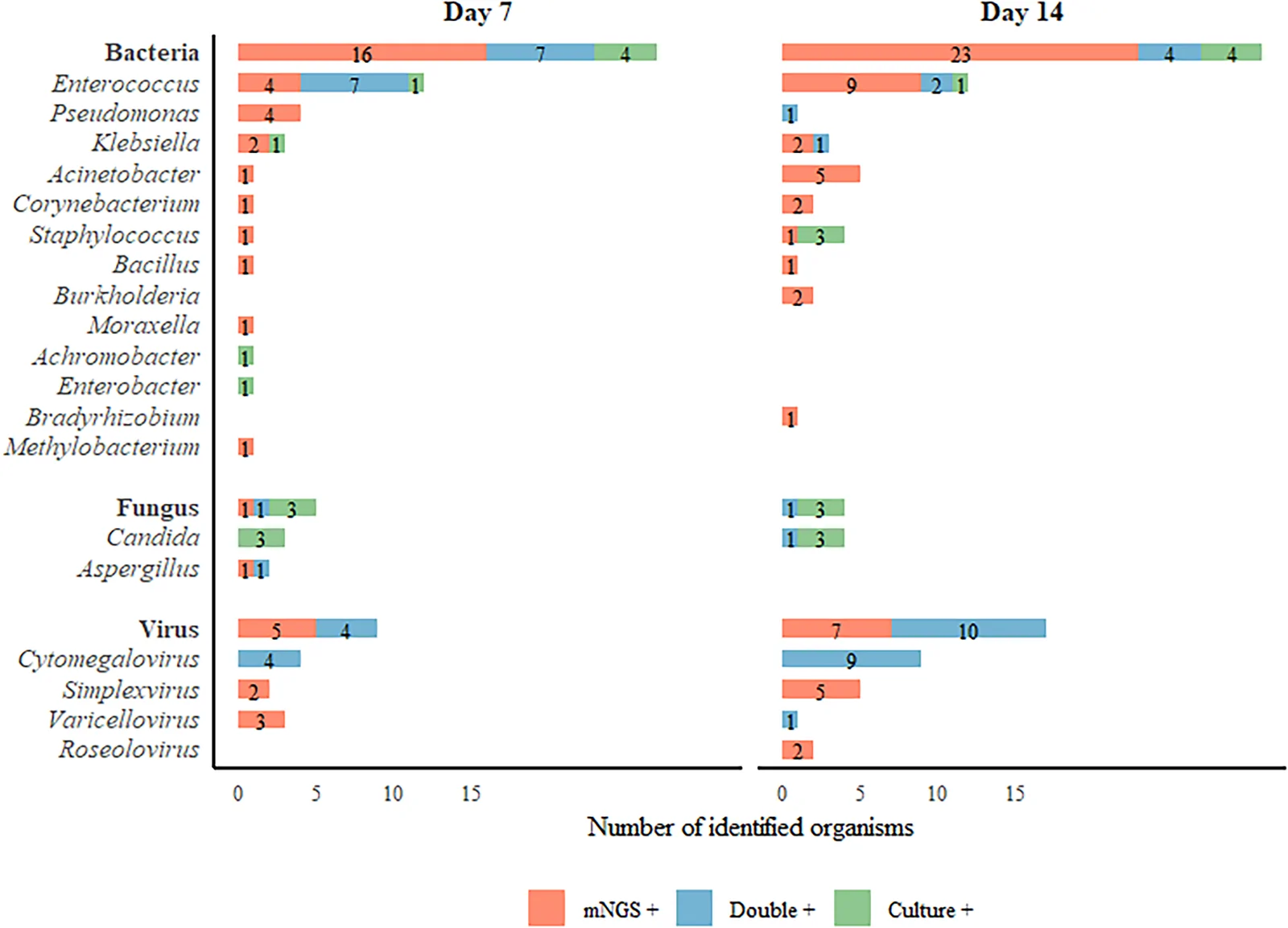
Distribution of microorganisms detected using mNGS and blood culture in liver transplantation recipients. mNGS results for *Aspergillus* are compared with those of the *Aspergillus* galactomannan assay. Viral detection is compared with PCR results from blood samples; positive cases only using PCR testing are not shown. mNGS, metagenomic next-generation sequencing; PCR, polymerase chain reaction.

mNGS detected additional viral pathogens, including herpes simplex virus and varicella-zoster virus, which were not routinely tested. CMV detection using mNGS was confirmed using quantitative PCR. The CMV PCR results according to mNGS CMV detection are summarized in **Supplementary Table S3**, and a detailed list of all microorganisms identified using mNGS is provided in **Supplementary Table S4**. Clinical correlation suggested possible infection in a subset of patients (**Supplementary Table S5**).

### 3.4 Analysis of discordant results between mNGS and blood culture

Thirteen discordant mNGS-negative and blood culture-positive cases were identified (**Table 2**). Two cases (15.4%) were categorized as contamination/colonization, five (38.5%) as weak positives, and six (46.2%) as not detected. Among the six non-detected cases, four were caused by Candida species, one by Enterococcus faecium, and one by *Achromobacter* spp.

**Table 2:** Results of the analysis of discordant cases with mNGS-negative and blood culture-positive results.

		Possible explanation		
Microorganism	Count	Contamination/ colonization	Weak positive	Not detected
*Candida* spp.	6	0	2	4^a)^
CoNS	2	2	0	0
*Klebsiella pneumoniae*	1	0	1	0
*Enterococcus faecium*	2	0	1	1^a)^
*Klebsiella aerogenes*	1	0	1	0
*Achromobacter* spp.	1	0	0	1

a) Two *Candida* and one *Enterococcus faecium* infections are identified only from catheter blood cultures and not from peripheral blood cultures.

mNGS, metagenomic next-generation sequencing; CoNS, coagulase-negative staphylococci.

### 3.5 Cross-validation of metagenomic fungal calls

The primary Kraken2-custom pipeline achieved high precision for fungal profiling, confirming 92.9% (13/14) of its fungal detections by direct alignment (**Supplementary Figure S1**). The auxiliary pipelines behaved as expected: EukDetect exhibited perfect precision (100.0%, 9/9 confirmed) but low relative sensitivity (7.3%), whereas Kaiju captured 100.0% of all alignment-confirmed fungi but had a lower precision of 89.9%. Across the cohort, 138 candidate calls (sample × species pairs) were adjudicated by targeted BWA-MEM alignment, resulting in 124 (89.9%) confirmed detections and 14 (10.1%) refuted calls (**Supplementary Figure S2**). Multi-pipeline concordance analysis demonstrated that 2-pipeline consensus confirmed 94.7% (18/19) of detections, and 3-pipeline consensus reached 100.0% (2/2) confirmation. Analysis of the overlap across methods (**Supplementary Figure S3**) showed that Kaiju captured nearly all candidate reads, with Kraken2 and EukDetect calls nested almost entirely within the Kaiju detection set.

### 3.6 Clinical characteristics of day 7 mNGS-positive patients by day 14 mNGS results

Among 160 patients who underwent mNGS testing on both days 7 and 14, 22 were mNGS-positive on day 7 (**Supplementary Table S6**). Of these, 15 remained positive on day 14, with the same organism persisting in 13 (86.7%). Patients with persistent mNGS positivity had higher Sequential Organ Failure Assessment scores and bilirubin and C-reactive protein levels, although differences in only total bilirubin on day 7 reached statistical significance. The serum immunoglobulin G levels were lower, and hypogammaglobulinemia was more frequent in this group, although these differences were not significant.

## 4 Discussion

In the present study, mcfDNA-based mNGS was performed during the early post-transplantation period to evaluate its diagnostic accuracy and clinical utility. Notably, concordance with the blood cultures was low at 35.0% on day 7 and 14.3% on day 14. However, when the culture results from other clinically reasonable specimens were considered, the overall concordance increased to 80.0% on day 7 and 71.4% on day 14, indicating a clinically acceptable level of agreement. Furthermore, mNGS identified pathogens not detected using the blood culture method, highlighting its ability to detect etiological organisms missed by conventional methods. mNGS also detected viral infections that would otherwise require targeted PCR testing, suggesting its clinical value in early post-LT evaluation.

mNGS has demonstrated higher sensitivity than conventional culture-based methods for diagnosing infectious diseases. Miao et al. (2018) reported a sensitivity of 50.7% for mNGS and 35.2% for culture in patients with clinically suspected infections, with mNGS showing a high positive predictive value of 91.2%. In patients with prior antibiotic exposure, the sensitivity of mNGS was significantly higher than that of culture alone (52.5% vs. 34.2%). Similarly, Li et al. (2024) reported superior diagnostic performance of mNGS compared with culture in patients with suspected infections, with higher positivity rates observed for mNGS (51.9% vs. 33.2%). Furthermore, Huang et al. (2022) reported a significantly higher pathogen positivity rate with mNGS than with traditional methods in donors who were undergoing deceased LT (64.3% vs. 28.6%). Notably, the same causative pathogens were detected in recipients via mNGS but remained undetected using traditional methods.

In the current study, concordance between mNGS and blood culture was low when compared directly, likely reflecting the higher sensitivity of mNGS and prior antibiotic exposure in most patients (Driscoll et al., 2017; Li et al., 2024; Miao et al., 2018). When cultures from other clinically relevant specimens were considered, the concordance between mNGS and conventional cultures increased, suggesting that mNGS more accurately identifies clinically meaningful pathogens. Furthermore, among patients who were mNGS-positive on post-LT day 7, the detection of the same microorganism in 86.7% of cases on day 14 of mNGS analysis indicated good reproducibility of the assay. Additionally, mNGS positivity was associated with greater clinical severity and a higher mortality, suggesting that mNGS results are closely related to the clinical status of LT recipients. Taken together, mNGS may offer higher sensitivity and better reproducibility for pathogen detection than blood culture in the early post-LT period.

The high analytical sensitivity of mNGS and its hypothesis-free, unbiased approach enabled the detection of microorganisms not identified using conventional diagnostic methods. Bacteria were detected exclusively using mNGS in 59.3% and 74.2% of the cases on post-LT days 7 and 14, respectively. Confirmatory PCR was not performed in many cases, reflecting the inherent limitation of conventional diagnostic approaches that rely on clinical suspicion to prompt targeted testing. Similarly, mNGS outperforms traditional methods in detecting *Mycobacterium tuberculosis*, viruses, anaerobes, and fungi (Miao et al., 2018). These findings highlight the utility of the hypothesis- and culture-independent nature of mNGS in identifying difficult-to-detect pathogens (Elbehiry and Abalkhail, 2025).

Viral infections other than CMV are less well-characterized, with limited and variable prophylactic strategies across countries (Besombes et al., 2025; Pedersen and Seetharam, 2014). These viral infections may arise after LT and present with mucocutaneous manifestations, which may be attenuated under immunosuppressive therapy, leading to under-recognition (Herrero et al., 2004; Pedersen and Seetharam, 2014; Phan et al., 2018; Wilck and Zuckerman, 2013). In our study, mNGS identified unexpected viral infections, and a retrospective review suggested that skin manifestations occurred in most patients despite the absence of diagnostic testing. Hence, viral infections after LT may not be adequately recognized or appropriately treated, and mNGS may serve as a useful adjunctive diagnostic tool. However, the lack of antiviral treatment in many cases raises the possibility that some detected viruses may represent bystanders rather than true pathogens, and their clinical significance warrants further investigation.

Despite the several advantages of mNGS, careful consideration is needed regarding its use in real-world clinical practice. Although mNGS demonstrates high sensitivity, this characteristic may also represent a limitation, as it can detect dead or dying pathogens, thereby complicating interpretation and potentially leading to unnecessary antimicrobial use (Elbehiry and Abalkhail, 2025). The high cost of testing is another important consideration. Therefore, rather than universal implementation after LT, selective use of mNGS in situations where the expected diagnostic benefit is high may be a more appropriate strategy. The low positivity rate observed in our study also supports this approach. Nevertheless, in our cohort, many mNGS-positive patients were already receiving broad-spectrum antimicrobial therapy at the time of mNGS testing, which likely limited the potential for mNGS to trigger unnecessary new antimicrobial treatment. Accordingly, mNGS may be selectively used as a complementary diagnostic tool to help identify rare or unexpected pathogens and support diagnosis in clinically ambiguous situations. Additionally, mNGS may facilitate early antibiotic de-escalation in patients already receiving broad empirical antimicrobial therapy. Future studies are needed to evaluate how mNGS influences antimicrobial modification and infection-related outcomes in LT recipients in real-world clinical practice.

Our validation study demonstrates that while metagenomic cfDNA profiling offers high precision for fungal pathogens at the species level, relying on a single taxonomic classifier introduces severe diagnostic blind spots. Kraken2 achieved a high precision of 92.9% (13/14 confirmed) for the species it successfully called, validating the biological authenticity of its positive detections. However, it exhibited a low relative sensitivity of only 10.5% (13/124) in our cohort, failing to detect several fungi, including *Candida* spp., *Nakaseomyces glabrata*, *Aspergillus niger*, and *Malassezia arunalokei*. All of these missed detections were identified by Kaiju using translated protein matching. These findings highlight the limitations of relying on a single classifier for fungal surveillance and support a multi-classifier consensus framework to balance diagnostic sensitivity and specificity. Nevertheless, additional fungal detections should be interpreted cautiously, as alignment-confirmed fungal DNA detection does not necessarily indicate active fungal infection or clinical pathogenicity. Further studies are needed to establish the clinical utility of the additional detections provided by Kaiju.

This study has some limitations. mNGS was performed in LT recipients, regardless of clinical signs or symptoms of infection; therefore, the positivity rate may be higher in settings where infection is clinically suspected. Although microbial DNA detection using mNGS does not invariably indicate active infection, its clinical relevance is supported by the high concordance with a culture from other clinical specimens and the worse outcomes among mNGS-positive patients. Additionally, because broad-spectrum antibiotics were administered in most cases at the time of blood culture collection, the difference in sensitivity between mNGS and blood cultures may have been overestimated. Owing to the retrospective nature of the study, we were unable to assess whether treatments guided by the organisms detected using mNGS were associated with improved clinical outcomes. Further, the cost-effectiveness and turnaround time of mNGS testing were not evaluated here. Detection of representative antimicrobial resistance genes using mNGS was also not validated. Kraken2 showed limited sensitivity for fungal detection at the species level; therefore, negative results cannot reliably exclude fungal infection. Finally, a universal RPTM ≥5 threshold was applied to all microbial candidates. Differentiated cut-off values, such as RPTM ≥3 for viruses and RPTM ≥8 for bacteria and fungi, may more effectively balance diagnostic sensitivity and specificity in plasma samples (H. Chen et al., 2024a; Y. Chen et al., 2024b; Miller et al., 2019).

## 5 Conclusion

Plasma mcfDNA mNGS shows substantial concordance with conventional microbiological testing when the results from clinically relevant specimens are considered. Moreover, mNGS detects additional pathogens not identified during routine diagnostic testing. Therefore, mNGS may serve as a useful adjunct for early pathogen detection and the clinical assessment of LT recipients. Nevertheless, further studies are needed to determine whether mNGS-guided management can improve clinical outcomes after LT.

## Data Availability

The host-depleted sequencing reads supporting this study are available in the European Nucleotide Archive (ENA) under accession number PRJEB127648 (https://www.ebi.ac.uk/ena/browser/view/PRJEB127648), with sample metadata recorded in accordance with the GSC MIxS host-associated checklist. The de-identified clinical data supporting the conclusions of this article are available from the corresponding author upon reasonable request, subject to applicable institutional and ethical requirements. Requests to access the datasets should be directed to SJ, jsj@yuhs.ac.
