# Supplementary material for: Plasma microbial cell-free DNA metagenomic sequencing for early detection of infection after liver transplantation: a prospective cohort study
Source: Front Cell Infect Microbiol. 2026 Oct 15;16:1930701. doi: 10.3389/fcimb.2026.1930701 (PMC13716750; doi:10.3389/fcimb.2026.1930701)
Supplement: Supplementary file 1 [file Supplementaryfile1.docx]

***Supplementary Material***

**1 Supplementary Data**

**1.1 Supplementary Methods S1. Immunosuppression protocol**

Most patients received basiliximab (20 mg on post-transplantation days 0 and 4) for induction immunosuppression. Patients undergoing ABO-incompatible living donor liver transplantation received desensitization therapy (including rituximab and plasmapheresis) according to the standard protocol at our institution.

Maintenance immunosuppressive therapy involved tacrolimus, prednisolone, mycophenolate mofetil (MMF), and everolimus. Tacrolimus doses were adjusted to maintain a target trough concentration of 6–10 ng/mL during the first month after transplantation and were gradually reduced to 3–8 ng/mL thereafter. MMF was initiated at a dose of 1.0–1.5 g/day, and this was subsequently adjusted to minimize adverse events, such as neutropenia, cytomegalovirus viremia, or severe infection. Everolimus is usually initiated 4 weeks after transplantation, replacing MMF in patients with hepatocellular carcinoma or renal insufficiency.

**1.2 Supplementary Methods S2. Detailed definitions of concordance**

Concordance between metagenomic next-generation sequencing (mNGS) and conventional diagnostic tests was assessed by comparing the detected organisms at corresponding time points. For bacterial pathogens, mNGS findings were compared with blood culture results obtained at the same time points. Organisms isolated from only a single bottle of the blood culture pair were included in the concordance assessment. For viral pathogens, mNGS results were compared using polymerase chain reaction testing. For fungal pathogens, concordance was assessed against the blood culture results. For *Aspergillus* species, the *Aspergillus* galactomannan antigen test was used for comparison.

**2 Supplementary Tables**

**Supplementary Table S1. Detailed clinical data and possibility of bacteremia in liver transplantation recipients with positive mNGS and negative cultures 7 days post-transplantation**

| **Case** | **Sex/Age (years)** | **mNGS** | **cfDNA, pg/mL** | **BC performed** | **Possible bacteremia** | **Related culture results** | **Clinical diagnosis** | **Prior active antibiotics** |  |
| --- | --- | --- | --- | --- | --- | --- | --- | --- | --- |
| 1 | M/54 | *Enterococcus* | 138,030 | - | + | D-6 Blood: EFA  D+3 Peritoneal: EFA | IAI, pneumonia | + |  |
| 2 | F/54 | *Enterococcus* | 258,000 | + | + | D-3 Peritoneal: EFA | IAI | + |  |
| 3 | F/51 | EFA | 47,730 | + | + | D+0 Peritoneal: EFA  D+2 Blood: EFA | IAI | - |  |
| 4 | M/67 | EFA | 52,030 | + | + | D-3 Blood: EFA D+0 Pleural, peritoneal: EFA | IAI, pneumonia | + |  |
| 5 | M/58 | *Klebsiella* | 110,510 | + | + | D-3 Sputum: KPN | IAI, Pneumonia | + |  |
| 6 | M/62 | *Klebsiella* | 90,300 | - | + | D-3 Blood: KPN  D-2, D+1 Peritoneal KPN | IAI | + |  |
| 7 | F/47 | *Pseudomonas* | 402,050 | + | + | D+1 Sputum, peritoneal: PsA | IAI, pneumonia | + |  |
| 8 | M/61 | *Acinetobacter, Corynebacterium,*  *Pseudomonas, Staphylococcus* | 10,791 | + | + | D+6 Peritoneal PsA | IAI | + |  |
| 9 | M/67 | *Bacillus* | 30,315 | + | + | D+6 H/V Bacillus | IAI | + |  |
| 10 | M/56 | *Pseudomonas* | 3,461,500 | + | - |  | IAI | + |  |
| 11 | M/57 | *Methylobacterium* | 11,451 | - | - |  | None | + |  |
| 12 | F/57 | *Pseudomonas* | 32,835 | - | - |  | IAI | - |  |
| 13 | M/46 | *Moraxella* | 42,500 | - | - |  | None | + |  |

mNGS, metagenomic next-generation sequencing; cfDNA, cell-free DNA; BC, blood culture; EFA, *Enterococcus faecium*; KPN, *Klebsiella pneumoniae*; PsA, *Pseudomonas aeruginosa*; H/V, Hemovac; IAI, intra-abdominal infection; M, male; F, female

**Supplementary Table S2. Detailed clinical data and the possibility of bacteremia in liver transplantation recipients with positive mNGS and negative cultures 14 days post-transplantation**

| **Case** | **Sex/Age (years)** | **mNGS** | **cfDNA, pg/mL** | **BC performed** | **Possible bacteremia** | **Related culture results** | **Clinical diagnosis** | **Prior active antibiotics** |  |
| --- | --- | --- | --- | --- | --- | --- | --- | --- | --- |
| 1 | F/54 | EFA | 23,865 | + | + | D-4 Blood: EFA  D+1 Peritoneal: EFA | IAI | + |  |
| 2 | F/63 | EFA | 22,575 | + | + | D-4 Blood EFA D-1 Peritoneal: EFA | IAI | + |  |
| 3 | F/51 | EFA | 50,310 | + | + | D-5 Blood: EFA D+0 Peritoneal: EFA | IAI | + |  |
| 4 | F/54 | EFA | 225,750 | + | + | D-10 Peritoneal: EFA | IAI | + |  |
| 5 | M/67 | EFA | 10,750 | + | + | D-10 Blood: EFA D-10 Pleural, peritoneal: EFA D-3 Peritoneal: EFA | IAI | + |  |
| 6 | M/59 | EFA, *Acinetobacter* | 39,345 | + | + | D-3 Blood: AB, EFA D+0 Peritoneal AB | IAI | + |  |
| 7 | M/64 | EFA, *Corynebacterium* | 21,715 | + | + | D-9 Peritoneal: Corynebacterium D-6 Blood: EFA | IAI | + |  |
| 8 | M/45 | *Enterococcus, Acinetobacter* | 12,040 | + | + | D-4 Blood: EFA D+0 Peritoneal: EFA, AB | IAI | + |  |
| 9 | M/57 | *Enterococcus, Burkholderia* | 6,600 | - | + | D-6 Urine: EFA | IAI | - |  |
| 10 | M/62 | *Klebsiella* | 89,870 | - | + | D-10 Blood: KPN  D+0 Peritoneal: KPN | IAI | + |  |
| 11 | M/67 | *Bacillus* | 28,380 | + | + | D-14 H/V: Bacillus | IAI | + |  |
| 12 | F/59 | *Corynebacterium* | 26,660 | + | + | D-6, D-3 Blood: Corynebacterium | IAI | + |  |
| 13 | M/54 | *Klebsiella* | 32,300 | + | - | D-1 Peritoneal: PsA | None | + |  |
| 14 | F/68 | *Acinetobacter, Staphylococcus* | 18,317.5 | + | - |  | IAI, pneumonia | + |  |
| 15 | M/48 | *Burkholderia* | 12,540 | + | - |  | None | + |  |
| 16 | M/50 | *Acinetobacter* | 16,254 | + | - |  | IAI | + |  |
| 17 | M/60 | *Bradyrhizobium* | 20,955 | - | - |  | None | NA |  |
| 18 | M/53 | *Acinetobacter* | 27,520 | - | - |  | None | + |  |

mNGS, metagenomic next-generation sequencing; cfDNA, cell-free DNA; BC, blood culture; EFA, *Enterococcus faecium*; KPN, *Klebsiella pneumoniae*; AB, *Acinetobacter baumannii*; PsA, *Pseudomonas aeruginosa*; H/V, Hemovac; IAI, intra-abdominal infection; M, male; F, female

**Supplementary Table S3. Comparison of cytomegalovirus detection using mNGS and quantitative PCR testing in liver transplantation recipients**

|  | **Post-LT, day 7** | | |  | **Post-LT, day 14** | | |
| --- | --- | --- | --- | --- | --- | --- | --- |
| **Variable** | **mNGS CMV (–)**  **(n = 175)** | **mNGS**  **CMV (+)**  **(n = 4)** | ***P*-value** |  | **mNGS CMV (–)**  **(n = 163)** | **mNGS**  **CMV (+)**  **(n = 11)** | ***P*-value** |
| CMV qPCR, copies/mL | 0.0 (0.0–0.0) | 36,950.0 (25,300.0–728,200.0) | <0.001 |  | 0.0 (0.0–66.0) | 5,770.0 (1,337.5–65,650.0) | <0.001 |
| Undetectable | 130 (77.8) | 0 (0.0) |  |  | 102 (64.6) | 0 (0.0) |  |
| <10^2^ | 10 (6.0) | 0 (0.0) |  |  | 29 (18.4) | 1 (9.1) |  |
| 10^2^–10^3^ | 18 (10.8) | 0 (0.0) |  |  | 21 (13.3) | 2 (18.2) |  |
| 10^3^–10^4^ | 8 (4.8) | 0 (0.0) |  |  | 5 (3.2) | 4 (36.4) |  |
| 10^4^–10^5^ | 1 (0.6) | 3 (75.0) |  |  | 1 (0.6) | 1 (9.1) |  |
| ≥10^5^ | 0 (0.0) | 1 (25.0) |  |  | 0 (0.0) | 3 (27.3) |  |

mNGS, metagenomic next-generation sequencing; PCR, polymerase chain reaction; LT, liver transplantation; CMV, cytomegalovirus; qPCR, qualitative polymerase chain reaction

**Supplementary Table S4. Identified microorganisms using mNGS in liver transplantation recipients**

|  |  | **Total**  **(n = 64)** | **mNGS, D7**  **(n = 26)** | **mNGS, D14**  **(n = 38)** | ***P*-value** |
| --- | --- | --- | --- | --- | --- |
| **Bacteria** |  | 41 (64.1) | 20 (76.9) | 21 (55.3) | 0.076 |
| *Enterococcus* |  | 22 (34.4) | 11 (42.3) | 11 (28.9) | 0.269 |
| *E. faecium* |  | 15 (23.4) | 7 (26.9) | 8 (21.1) | 0.586 |
| *Acinetobacter* |  | 6 (9.4) | 1 (3.8) | 5 (13.2) | 0.209 |
| *Pseudomonas* |  | 5 (7.8) | 4 (15.4) | 1 (2.6) | 0.062 |
| *Klebsiella* |  | 5 (7.8) | 2 (7.7) | 3 (7.9) | 0.976 |
| *Corynebacterium* |  | 3 (4.7) | 1 (3.8) | 2 (5.3) | 0.792 |
| *Staphylococcus* |  | 2 (3.1) | 1 (3.8) | 1 (2.6) | 0.784 |
| *Bacillus* |  | 2 (3.1) | 1 (3.8) | 1 (2.6) | 0.784 |
| *Burkholderia* |  | 2 (3.1) | 0 (0.0) | 2 (5.3) | 0.235 |
| *Moraxella* |  | 1 (1.6) | 1 (3.8) | 0 (0.0) | 0.223 |
| *Bradyrhizobium* |  | 1 (1.6) | 0 (0.0) | 1 (2.6) | 0.404 |
| *Methylobacterium* |  | 1 (1.6) | 1 (3.8) | 0 (0.0) | 0.223 |
| **Virus** |  | 28 (43.8) | 10 (38.5) | 18 (47.4) | 0.481 |
| Cytomegalovirus |  | 15 (23.4) | 4 (15.4) | 11 (28.9) | 0.208 |
| Simplexvirus |  | 7 (10.9) | 2 (7.7) | 5 (13.2) | 0.491 |
| Varicellovirus |  | 5 (7.8) | 4 (15.4) | 1 (2.6) | 0.062 |
| Roseolovirus |  | 2 (3.1) | 0 (0.0) | 2 (5.3) | 0.235 |
| **Fungus** |  | 3 (4.7) | 2 (7.7) | 1 (2.6) | 0.347 |
| *C. glabratus* |  | 1 (1.6) | 0 (0.0) | 1 (2.6) | 0.404 |
| *Aspergillus* |  | 2 (3.1) | 2 (7.7) | 0 (0.0) | 0.082 |
| **Parasite**^a)^ |  | 1 (1.6) | 0 (0.0) | 1 (2.6) | NA |

^a)^*Enterobius vermicularis* is identified.

mNGS, metagenomic next-generation sequencing

**Supplementary Table S5. Detailed clinical data of liver transplantation recipients with viral pathogens identified using mNGS, excluding cytomegalovirus**

| **Case** | **Sex/Age** | **Day** | **mNGS** | **cfDNA, pg/mL** | **PCR performed** | **Antiviral treatment** | **Clinical presentation** |  |
| --- | --- | --- | --- | --- | --- | --- | --- | --- |
| 1 | F/62 | 7 | Simplexvirus | 29,455 | - | - | Oral mucositis |  |
| 2 | M/56 | 7, 14 | Simplexvirus | 105,700 | - | Ganciclovir for CMV | Oral mucositis, purpuric patches |  |
| 3 | M/75 | 7 | Varicellovirus | 42,355 | - | Acyclovir for shingles | Skin rash, herpes zoster |  |
| 4 | F/61 | 7 | Varicellovirus | 402,050 | - | Ganciclovir for CMV | Skin purpuric papules |  |
| 5 | M/60 | 7 | Varicellovirus | 110,510 | - | - | Multiple erosive to ulcerative lesions on the buttocks |  |
| 6 | M/64 | 14 | Simplexvirus | 5,858 | - | - | Skin ulcer, oral ulcer |  |
| 7 | F/72 | 14 | Simplexvirus | 37,195 | - | - | Oral erosion |  |
| 8 | M/58 | 14 | Simplexvirus | 44,720 | - | - | - |  |
| 9 | M/51 | 14 | Simplexvirus | 33,250 | - | - | Ulcerative erosions on the buttocks |  |
| 10 | M/55 | 14 | Varicellovirus | 51,600 | Blood, skin lesion PCR(+) | Acyclovir for disseminated zoster | Disseminated zoster |  |
| 11 | M/61 | 14 | Roseolovirus (HHV6B) | 75,680 | - | - | Abdominal abrasion or ulcer |  |
| 12 | M/54 | 14 | Roseolovirus (HHV6B) | 32,300 | - | - | Perioral multiple vesicles, purpuric macules on the abdomen |  |

mNGS, metagenomic next-generation sequencing; PCR, polymerase chain reaction; CMV, cytomegalovirus; HHV, human herpesvirus; M, male; F, female; cfDNA, cell-free DNA

**Supplementary Table S6. Comparison of disease severity in patients positive for mNGS on day 7 by mNGS positivity on day 14**

|  |  | **mNGS, D14 (–)**  **(n = 7)** | **mNGS, D14 (+)**  **(n = 15)** | ***P*-value** |
| --- | --- | --- | --- | --- |
| Same organism identified |  | - | 13 (86.7)^a)^ | NA |
| MELD score |  | 18.6 ± 12.7 | 25.9 ± 10.8 | 0.177 |
| Inotropics use, D7 |  | 2 (28.6) | 6 (40.0) | 0.604 |
| Inotropics use, D14 |  | 2 (28.6) | 5 (33.3) | 0.823 |
| Mechanical ventilator, D7 |  | 2 (28.6) | 6 (40.0) | 0.604 |
| Mechanical ventilator, D14 |  | 2 (28.6) | 4 (26.7) | 0.926 |
| SOFA, D7 |  | 6.7 ± 6.1 | 9.8 ± 3.4 | 0.138 |
| SOFA, D14 |  | 3.0 (2.5–7.5) | 6.0 (3.5–9.0) | 0.415 |
| ∆SOFA |  | −1.0 ± 1.8 | −3.0 ± 3.2 | 0.145 |
| CRP, D7 |  | 17.7 (9.6–58.3) | 40.6 (20.2–65.1) | 0.503 |
| CRP, D14 |  | 63.6 ± 79.4 | 91.3 ± 47.0 | 0.314 |
| ∆CRP |  | 12.1 ± 37.7 | 41.3 ± 43.6 | 0.143 |
| T. bilirubin, D7 |  | 2.0 (1.5–2.9) | 5.9 (3.6–9.5) | 0.044 |
| T. bilirubin, D14 |  | 1.8 (1.1–5.0) | 3.8 (2.7–6.3) | 0.481 |
| ∆T. bilirubin |  | −0.5 (–1.4 to 1.2) | −0.9 (–1.9 to 0.6) | 0.698 |
| IgG, D7 |  | 856.0 (708.0–998.5) | 600.0 (552.0–779.0) | 0.205 |
| IgG, D14 |  | 782.0 (627.5–1160.5) | 575.0 (451.0–771.0) | 0.224 |
| ∆IgG |  | −190.0 (–241.0 to –31.0) | −131.0 (–165.0 to 41.0) | 0.351 |
| HGG, D7 |  | 2 (28.6) | 8 (61.5) | 0.160 |
| HGG, D14 |  | 3 (42.9) | 10 (71.4) | 0.204 |

^a)^Identified organisms include seven *Enterococci*, one *Klebsiella*, one *Bacillus*, three CMV, and one HSV.

mNGS, metagenomic next-generation sequencing; MELD, Model for End-stage Liver Disease; SOFA, Sequential Organ Failure Assessment; CRP, C-reactive protein; IgG, immunoglobulin G; HGG, hypogammaglobulinemia; CMV, cytomegalovirus; HSV, herpes simplex virus; T., total; NA, not applicable

**Supplementary Figure S1. Per-method classification performance at the fungal species level,** (A) Precision: The proportion of species-level calls generated by each classification pipeline that were confirmed by high-stringency BWA-MEM alignment to eukaryotic reference genomes. (B) Relative sensitivity: The proportion of all alignment-confirmed calls in the cohort detected by each classifier (relative to the union of the three methods). Error bars indicate Wilson score 95% confidence intervals.

**Supplementary Figure S2. Genus-level summary of validation results and alignment-based confirmation.** Genus-level metagenomic validation results were aggregated from species-level calls (where any confirmed species confirms the genus in that sample). Horizontal bars represent the total number of patient samples in which each genus was called, split into confirmed (green/solid) and refuted (orange/hatched) by BWA-MEM mapping. Bar colors indicate the union of classification methods that generated the species-level calls for that genus. Numbers next to the bars denote the total sample counts.

s
